# N-3 Polyunsaturated Fatty Acids as a Nutritional Support of the Reproductive and Immune System of Cattle—A Review

**DOI:** 10.3390/ani13223589

**Published:** 2023-11-20

**Authors:** Julia Fabjanowska, Edyta Kowalczuk-Vasilev, Renata Klebaniuk, Szymon Milewski, Hıdır Gümüş

**Affiliations:** 1 Institute of Animal Nutrition and Bromatology, University of Life Sciences in Lublin, 20-950 Lublin, Poland; julia.fabjanowska@up.lublin.pl (J.F.); renata.klebaniuk@up.lublin.pl (R.K.); szymon.milewski@up.lublin.pl (S.M.); 2 Department of Animal Nutrition and Nutritional Diseases, Faculty of Veterinary Medicine, University of Burdur Mehmet Akif Ersoy, 15030 Burdur, Türkiye; hgumus@mehmetakif.edu.tr

**Keywords:** fatty acids, omega 3, cattle, nutrition, reproduction, immunity

## Abstract

**Simple Summary:**

This review focuses on two crucial aspects of cattle breeding: the reproduction of dairy cows and the health status and body condition of cows during the periparturient period, as well as the immunity of newborn calves. The energetic importance of fat in ruminant nutrition and lipids metabolism in the rumen, the regulatory role of n-3 fatty acids in the reproduction of ruminants, their importance in fetus developmental programming and embryo development, as well as the role of n-3 fatty acids a key component in the building of the immunity of newborn calves, are discussed.

**Abstract:**

This paper focuses on the role of n-3 fatty acids as a nutrient crucial to the proper functioning of reproductive and immune systems in cattle. Emphasis was placed on the connection between maternal and offspring immunity. The summarized results confirm the importance and beneficial effect of n-3 family fatty acids on ruminant organisms. Meanwhile, dietary n-3 fatty acids supplementation, especially during the critical first week for dairy cows experiencing their peripartum period, in general, is expected to enhance reproductive performance, and the impact of its supplementation appears to be dependent on body condition scores of cows during the drying period, the severity of the negative energy balance, and the amount of fat in the basic feed ration. An unbalanced, insufficient, or excessive fatty acid supplementation of cows’ diets in the early stages of pregnancy (during fetus development) may affect both the metabolic and nutritional programming of the offspring. The presence of the polyunsaturated fatty acids of the n-3 family in the calves’ ration affects not only the performance of calves but also the immune response, antioxidant status, and overall metabolism of the future adult cow.

## 1. Introduction

Oils and fats of plant origin occupy an important place in global food production, accounting for about 79% of the total annual production [1]. Due to the increasing production potential of oilseed crops and the possibility of using them to improve nutritional value and optimize rations, thereby improving the quality of animal nutrition, they are gaining importance as ingredients of feed additives and concentrates [2,3]. This is also because they are a rich source of energy for the optimal functioning of the body’s organs and tissues [4,5,6], as their energy density is more than two times higher compared to other organic components [7]. Fat is an important supplement to the feed ration, contributing not only to energy concentration but also improving the absorption of fat-soluble vitamins (A, D, E, K) and increasing palatability and feed utilization [4].

**Figure 1:**
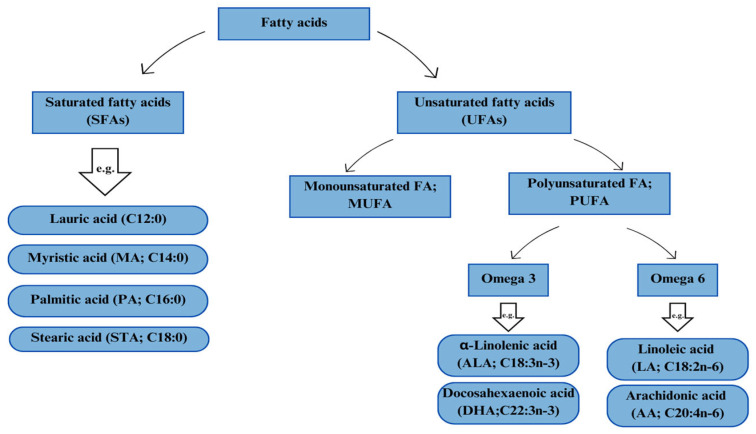
Classification of fatty acids.

However, lipid metabolism in the body is determined by the type of fatty acids (FAs) supplied with the feed. The lipid components supplemented in animal diets increase the concentration of biologically active fatty acids contained therein [8]. The role of fatty acids in the animal body shows a wide variety of effects that are determined primarily by the structure of these biologically active molecules [9]. The structure of fatty acids varies in terms of carbon chain length and the degree of saturation [10]. Fatty acids, due to their ability to be chemically modified, contribute to functionality and biological activity by making them the basis of many animal body processes [11]. They are found in cells, wherein they play a role in various functions including the construction of cell membranes, transport and absorption of nutrients, or production of hormones [12,13]. Based on their chemical structure and biochemical properties, fatty acids can be divided into two main groups. The first group is saturated fatty acids (SFAs), such as palmitic acid (PA; C16:0), lauric acid (C12:0), myristic acid (MA; C14:0), and stearic acid (STA; C18:0). Saturated fatty acids are mainly responsible for energy transport but also prevent damage to cell membranes caused by lipid oxidation reactions. Raphael and Sordillo [14] described in vitro studies in which saturated fatty acids (SFAs) directly activated pro-inflammatory signaling pathways and increased the expression of pro-inflammatory cytokines, mimicking the action of endotoxins. The second group is unsaturated fatty acids (UFAs), which contain one (monounsaturated FA; MUFA) or more (polyunsaturated FA; PUFA) double bonds in their structure. Polyunsaturated fatty acids (PUFAs) are an integral structural part of phospholipids. The classification of fatty acids is presented in Figure 1.

The crucial factor for ruminants is the amount of polyunsaturated fatty acids provided in a diet. Thus, this paper focuses on the role of n-3 family acids as a nutrient of pivotal importance for the proper course of many processes in the body. The presence of the polyunsaturated fatty acids of the n-3 family positively affects an animal’s body, namely, among other things, its growth and development, as well as the stabilization of its immune system [15,16]. N-3 fatty acids affect the activity of cell membranes and promote the interaction of enzymes by acting as intermediaries in enzymatic reactions. In addition, some FAs are the precursors for many biologically active substances, such as eicosanoids, including prostaglandins, leukotrienes, and thromboxanes. N-3 fatty acids can be used as substrates in the formation of anti-inflammatory lipid mediators and resolvins [17]. EPA (C20:5n-3) and DHA (C22:6n-3) show the potential to modulate the inflammatory response by inhibiting the production of pro-inflammatory cytokines [18]. The enzymes responsible for converting n-6 and n-3 FAs into these precursors (delta-6 desaturase, elongase-5, and delta-5 desaturase) and eicosanoid synthesis (cyclooxygenase and lipoxygenase) are the same regardless of the FA type, meaning that n-6 and n-3 FAs compete for eicosanoid synthesis. N-3 PUFAs act as ligands for transcription factors, which are responsible for regulating genes involved in metabolic and developmental processes [19]. Their action also affects the transcriptome, which is the complete set of all nucleic acid molecules in the cell, thus showing nutrigenomic effects [20]. This contributes to regulating metabolic processes occurring in the body and improving the health of ruminant animals [21,22]. In addition, they can modulate the expression of genes related to the inflammatory response, lipid metabolism, and DNA methylation [7]. Among the dietary supplements that are a good source of unsaturated fatty acids (UFAs) used in ruminant nutrition are vegetable and fish oils.

The state of knowledge on the effects of n-3 family fatty acids on body function and animal health is developing rapidly. At the same time, due to the complexity of the effects of fatty acids on the functioning of the ruminant organism, which, in addition to the positive effects on the body, can affect changes in the microbial activity of the rumen, and the impact of polyunsaturated acids on ruminants has been inaccurately described. This study aims to systematize the current state of knowledge on the role of n-3 acids in the proper functioning of the reproductive and immune systems, with nutritional support for cattle.

## 2. The Energetic Importance of Fat in Ruminant Nutrition

**Figure 2:**
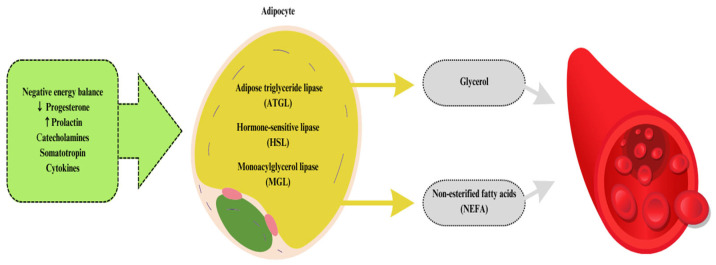
The process of lipolysis involved in fat mobilization.

The intensification of the rearing of polygastric animals, mainly dairy cattle, has contributed to an increased number of scientific reports on the impact of inadequately balanced rations in terms of animal energy requirements and the impact of supplementing ruminant diets with various natural energy sources [23]. The energy balance associated with the transition into lactation is a challenge for many breeders. The problem of energy deficiencies in dairy cows intensifies especially during the transition period [24]. During the periparturient period, cows reduce feed intake, which, along with increased energy requirements (necessary for lactogenesis and fetal growth) that exceed the available amount for the animal, contributes to a state of negative energy balance [25]. Dairy cows that do not have an adequate adaptive response to the negative energy balance may experience the onset of metabolic disorders, including subclinical ketosis. Moreover, energy deficits can also extend over a much longer period of milk production in cows [26]. The most common reason for worsening a negative energy balance is the insufficient energy value of feed intake compared to the requirement [27]. Ruminant animals living in the wild are also affected by this issue, as their daily dietary intake is characterized by a relatively low amount of fats, which also contributes to the energy deficit. In addition, during the transition period, there are changes in the hormonal economy in the body, which contribute to changes in the activity of body tissues by increasing lipolysis and decreasing lipogenesis. Lipolysis at the beginning of lactation is a genetically determined process (homeorhesis), while the enzymes involved in lipogenesis are regulated by energy intake (homeostasis) [28]. The result of the changes that have occurred is the metabolic mobilization of adipose tissue activated by, among others, catecholamines, cytokines, and growth hormones, leading to the breakdown of triacylglycerols present in adipocytes (fat cells) into glycerol and non-esterified fatty acids (NEFAs) to be released into the bloodstream (Figure 2) [29]. They are transported by various lipids, which include neutral lipids (NLs) and phospholipids (PLs) [25].

Neutral lipids consist of triglycerides, diglycerides, monoglycerides, and cholesterol esters. The fatty acid profile of individual lipids varies, e.g., linolenic acid is found in neutral lipids, while polyunsaturated fatty acids (PUFAs) are found in phospholipids. During lipolysis, circulating NEFAs released from adipose tissue enter the liver and can be utilized in four pathways: (1) they can be completely oxidized for energy in the Krebs cycle, (2) they are converted to beta-hydroxybutyrate (BHB), (3) they can be re-synthesized to triglycerides (TG), where they can either be exported by very low-density lipoproteins (VLDL) or stored in the liver, or (4) they can be used to synthesize milk fatty acids in the mammary gland [30]. The beta-oxidation of fatty acids occurs in the mitochondria of liver cells, and the resulting Acetyl CoA releases energy in the tricarboxylic fatty acid cycle [31,32]. Carnitine palmitoyltransferase-I (CPT-I) is a limiting enzyme for the transport of fatty acids into the mitochondria where beta-oxidation occurs [33,34]. In cows experiencing a state of negative energy balance, an increase in the activity of carnitine palmitoyltransferase I (CPT-I) is observed, as well as an upregulation of beta-oxidation [34]. When the mobilization of fatty acids from body tissues is very intense, the acetyl CoA generated through B-oxidation is converted into ketone compounds such as acetoacetate and B-hydroxybutyric acid [35]. The rate of formation of ketone bodies is not as energy-efficient as B-oxidation. In early lactation, however, ketogenesis is an important process because oxidized ketone compounds provide energy for vital organs [36,37]. Free fatty acids that do not undergo hepatic B-oxidation are re-esterified into triglycerides and enter the bloodstream as very low-density lipoproteins (VLDLs). The intensity of the re-esterification process increases in a negative energy balance (NEB) state. However, the rate of VLDL transport from the liver of cows is low due to the low synthesis capacity of proteins (apoprotein B) that control the rate of VLDL synthesis and secretion [38,39]. These mechanisms result in the accumulation of triglycerides in hepatocytes, leading to fat cow syndrome. Furthermore, cows with excessively high body condition scores (BCSs) in the early stages of the drying period mobilize fat tissue to a greater extent before parturition than those with normal conditions or low BCSs [40]. Reducing the severity of NEB and triglyceride accumulation in the liver is possible, for example, by shortening the dry (nonlactating) period. Elevated concentrations of NEFA result in a reduction in cell number, chemotactic capacity, phagocytic activity, and the oxidative burst activity of polymorphonuclear cells [41]. In addition, NEFA can lead to an increase in the number of reactive oxygen species and the breakdown of neutrophil granulocytes, a decrease in leukocyte proliferation and stimulation, and a decrease in the secretion of IgM and IFN-γ [41]. Higher concentrations of those metabolites are typical in dairy cows in a transition period; however, the low capacity of the animal body to break them down results in the risk of production parameter disorders and the health status of cattle.

During the periparturient period, a negative energy balance is, to some extent, a biological necessity due to high energy requirements with insufficient energy intake from feed. At the same time, hypoglycemia occurs as a result of the increased demand for glucose—the primary precursor for the synthesis of milk lactose, which controls milk volume by maintaining the osmolarity of milk. In addition, during the resulting negative energy balance (NEB), cows use the release of nutrients from their body tissues as a key mechanism to maintain lactation as compensation for reduced nutrient intake from their diet [42]. As a result of adipose tissue mobilization in animals, an increase in saturated and monounsaturated fatty acids can contribute to the compensation of the negative energy balance.

An increased ratio of unsaturated fatty acids (UFAs) to saturated fatty acids (SFAs) and the ratio of n-3 to n-6 fatty acids (PUFAs) can lead to biological membrane dysfunction and the onset of oxidative stress, which, in turn, can affect the health of dairy cows [42]. This is extremely important because fat mobilization can result in too much lipid peroxidation in the liver and free radical formation, which can escalate oxidative stress in the animal body [43]. Consequently, there is a great need for nutritional strategies to reduce the adverse effects of NEFA by mitigating fat mobilization and accelerating NEFA removal from the liver. An adequate supply of energy with the feed ration can reduce the severity of body fat mobilization to some extent [27]. Among these, we can highlight fatty acid supplementation, which has the effect of not only increasing the energy density of the diet but also mobilizing immune cells and the inflammatory response [44]. In addition, polyunsaturated free fatty acids (FFAs), such as n-3 fatty acids and conjugated linoleic acids, are fed to reduce "de novo" fatty acid synthesis in the udder and thus the TAG content of milk, which can have modest benefits for the overall energy balance.

Furthermore, an excessive proportion of SFA caused by fat mobilization processes in the cell membrane of white blood cells significantly affects immune dysfunction and the formation of an unregulated inflammatory response in early lactation. This is due to a change in the profile of fatty acids in cell membranes, resulting in a change in the synthesis of oxylipids (also known as oxylipins or eicosanoids), which are responsible for anti-inflammatory and antimicrobial responses [45]. Ling et al. [46] demonstrated the impact of the lipid mobilization process occurring during the transition period on the fetus. The offspring of cows excessively exposed to non-esterified fatty acids were characterized by an increased index of the body’s oxidative status, which is expressed as the ratio of reactive oxygen and nitrogen species to the total antioxidant potential. The mechanism explaining the relationship between prenatal exposure to high maternal NEFA concentrations in late gestation and increased oxidative stress in born calves is unknown. However, in vitro studies indicate that fatty acids can directly induce the production of reactive oxygen and nitrogen species (RONS) in cells and indirectly affect oxidative stress in the organism through the activation of metabolic inflammatory pathways [47]. Therefore, increasing the energy value of the diet, on the other hand, may have a beneficial effect in reducing the duration and severity of a negative energy balance condition while maintaining high milk yields in high-yielding dairy cows [48].

## 3. Lipid Metabolism in Rumen—Impact of Rumen Microbiota on FA Biohydrogenation and Transformation and the Role of By-Pass Fat

**Figure 3:**
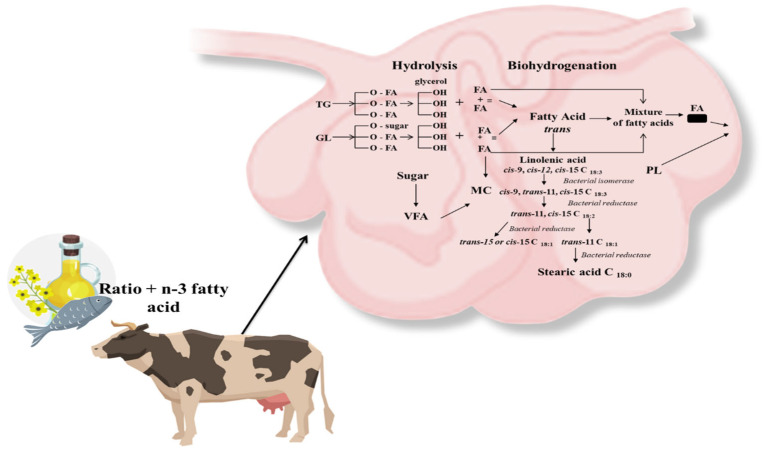
Lipid transformations in the rumen of dairy cows (based on Bauman et al. [50]). Abbreviations: TG—triglycerides; GL—glycolipids; FA—fatty acids; VFA—volatile fatty acids; PL—phospholipids; SFA—saturated fatty acid; MC—Microbial cells.

The diet of ruminant animals contains a small amount of fat. The daily rations contain no more than 6% lipids, half of which comes from forage and the rest is supplemental fat [22]. Unlike monogastric animals, due to chewing and rumen microbial activity, fatty acids reach further sections of the digestive tract in an altered form [49]. The intensive metabolic transformation of feed fats results in changes in the absorption of fatty acids and their synthesis in the body. The two main metabolic processes are the hydrolysis of esters to release free fatty acids (lipolysis) and the biohydrogenation of unsaturated fatty acids (Figure 3).

Several types of rumen bacteria are involved in this process [51]. The first step in rumen lipid metabolism is the lipolysis of feed lipids, which is the source of glyceride esters, consisting mainly of triacylglycerols from concentrates and galactolipids and phospholipids from forages, except for ensiled forages, from which free FAs are released by plant lipases. This process is determined by several factors, and the most important are rumen pH and the amount of fat in the daily ration, as too much of it decreases the activity of the lipolysis process [52,53]. As a result of this transformation, lipids entering the rumen are first converted by microbial lipases. Among the most active bacteria involved in lipolysis is *Anaerovibrio lipolytica*. However, their metabolites only affect triacylglycerols. In the case of galacto- and phospholipids, the most involved are lipases derived mainly from the bacteria of the genus *Butyrivibrio*. As a result of their action, ester bonds of complex lipids are hydrolyzed so that unsaturated fatty acids such as linoleic acid (cis-9, cis-12 C18:2), linolenic acid (cis-9, cis-12, cis-15 C18:3), or oleic acid (cis-9 C18:1) are released along with glycerol. The rate of their transformation increases as the degree of unsaturation increases [54]. FAs released by lipolysis are rapidly hydrogenated by the action of bacterial isomerases, followed by reductases. The isomerization process contributes to a change in the geometric configuration of the acids. Due to the displacement of the double bond and its transformation from the *cis* to the *trans* form, conjugated linoleic acids are formed. In addition, the formation of several isomers is also observed, among which the predominant fatty acid trans-11 stands out. The next step is the reduction in double bonds, which leads to the hydrogenation of the *trans* double bonds formed during isomerization. Those fatty acids are partially incorporated into the bacteria responsible for the biohydrogenation process, during which polyunsaturated fatty acids (n-3 or n-6) are converted to monounsaturated (MUFA) or saturated (SFA) acids. The feed-derived acids, i.e., linoleic acid: C18:2, n-6 (LA) and C 18:3, n-3 linolenic acid (LNA) undergo biohydrogenation to stearic acid: C18:0. This process also results in the formation of trans-11-vaccenic acid (TVA, C18:1) and conjugated linoleic acid (CLA C18:2, cis-9, trans-11), along with isomers that are absorbed in the small intestine [52]. Only a small proportion of unsaturated fatty acids can enter the lumen of the small intestine in an unchanged form, from which they are absorbed into the lymph in the jejunum [52]. The amount of vaccenic acid (VA, trans-C18:1) entering the duodenum of cows’ ranges from 20 to 140 g, which is about 10–20% of the amount of PUFA fatty acids in the daily ration [52].

The degree of biohydrogenation depends primarily on the type of fat, the length of incubation time in the rumen, as well as the composition of the rumen microbiota population. Bacteria such as *Butyrivibrio fibrisolvens*, *Eubacterium* spp., *Ruminococcus albus*, *Borrelia*, *Micrococcus*, and *Fusocillus* spp. are involved in this process [55]. The amount of long-chain unsaturated fatty acids affects the degree of the biohydrogenation of polyunsaturated fatty acids and decreases with an increase in saturated FAs [55]. It is also dependent on the size of the rumen: in small ruminants (goats and sheep), the degree of total biohydrogenation is lower [56]. The introduction of a fat supplement into the diet of ruminants, especially one containing polyunsaturated fatty acids, can, therefore, have a negative impact on the rumen fermentation process and ultimately affect total feed digestibility. The excessive supplementation of ruminant animals with fats may result in increased amounts of certain trans fatty acids in livestock products and a decrease in the fat and protein content of milk [57,58]. Regarding growing ruminants, the dietary inclusion of lipids must be restricted (to ca. 60 g/kg DM consumed) to avoid the impairment of the rumen function [59]. For this reason, the manipulation of ingredients in the ration abundant in PUFA fatty acids available to the rumen flora is limited [60]. Their effect depends on the amount of additive introduced, its form, such as raw or processed (extruded, micronized) oilseeds, and the composition of the diet [61,62].

Increasing the energy value of ruminant animal feed without reducing its digestibility is achieved through the use of appropriate additives in the form of specially formulated fats called rumen-protected fats, including calcium soaps, which provide an important safeguard against rumen biohydrogenation [63]. One of the main advantages of using protected fat is its high digestibility rate, which is as high as 85%. Protected fats entering the rumen remain in their original form. In addition, they are inert to the rumen environment; therefore, they do not cause changes in their fermentation processes and lower the activity of cellulolytic microorganisms. Protected fat can be fed in the form of oilseeds that have undergone previous thermal processes, most often extrusion and micronization. Heat treatment in the form of an extrusion process of, for example, flax seeds, with optimally selected parameters [64] can change the site of protein digestion from the rumen to the small intestine without adversely affecting total nutrient digestibility [61], thus contributing to a faster fat absorption process [65]. The addition of extruded flaxseed not only increases the energy concentration in the ration but contributes to improving reproductive rates in ruminant animals through the specific effects of n-3 fatty acids [66], as well as increasing the proportion of polyunsaturated fatty acids contained in the milk of cows [61]. Extrusion also increases the absorption rate of amino acids available to the intestine, increasing the pool of amino acids available for protein synthesis in the mammary glands [67]. The complete digestion of protected fat takes place in the small intestine through the action of pancreatic lipase, and unchanged unsaturated fatty acids are absorbed in the small intestine [68]. They provide a highly cost-effective alternative for starch-restricted feeds without reducing energy levels in the ration of ruminant animals and without compromising productivity. Behan et al. [69] confirm this, claiming that the use of protected fats also plays a significant role in improving animal performance without affecting the rumen microbiome.

## 4. Regulatory Role of N-3 Fatty Acids in Reproduction of Ruminants

In the reproductive process, ovulation plays a key role, enabling the mature oocyte to have the ability to fertilize [70]. The process of mammalian oocyte growth involves an important epigenetic process—reprogramming the maternal genome. This process involves changes in the cytoplasmic organelles, synthesis, and storage of mRNAs and proteins that are essential for the initial cell cycles of embryogenesis, the resumption and termination of meiosis, and epigenetic modifications [71]. Striving for optimal metabolic health in dairy cows is a key strategy to ensure adequate ovarian physiology and high-quality oocytes and embryos [72].

Fatty acids (FAs) play a key role in oocyte developmental programming [73]. The concentration of n-3 fatty acids in an organism has an impact on the maturation process and developmental competence of oocytes [74]. Introducing an appropriate diet containing n-3 PUFAs can have a positive impact on oocyte quality, maturation ability, and the probability of conception in cows. Studies conducted on Holstein heifers have confirmed the importance of fatty acids in oocytes and their impact on developmental programming [71].

Polyunsaturated fatty acids (PUFAs), by altering hormone and metabolite levels, can affect various levels of the hypothalamic–pituitary–ovarian axis and locally stimulate follicle growth. Studies confirm that altering fatty acid (FA) intake in the diet of dairy cows can affect the concentration of circulating metabolic hormones and ovarian steroids, which, in turn, can affect the development and capacity of ovarian follicles and the composition and secretion of reproductive tissues [75,76]. Acting as the endogenous stimulators of peroxisome proliferator-activated receptors (PPARs), n-3 fatty acids influence the regulation of sex steroid hormones involved in the processes of ovarian follicle growth and differentiation, oocyte maturation, and embryo development [77].

During the ovulation process, the mature oocyte is surrounded by ovarian follicles that gradually grow [78]. During the pre-ovulatory stage, the oocyte accumulates nutrients, mRNAs, proteins, and mitochondria and is surrounded by specialized hill cells and antral cavity fluid [79]. The oocyte’s ability to develop into a viable embryo after fertilization is acquired mainly during terminal growth and meiotic maturation. The LH signal is transmitted through the granulosa and thalamus cells, which triggers the resumption of meiosis and the completion of the nuclear maturation of the oocyte into the metaphase II (MII) stage of meiosis, preparing it for fertilization [80,81]. These rapid and synchronized events, such as the proliferation of granulosa layer cells, generation of the thalamic cell matrix, and segregation of chromosomes, are energy-intensive processes that require the production of sufficient ATP [82]. Fatty acids play a key role as an energy source for the oocyte. The oocyte takes up both exogenous lipids and synthesizes and stores endogenous lipids in the ooplasm [83]. Lipids have an important function, namely, signaling molecules in the regulatory mechanisms of oocyte maturation and competence acquisition [84]. Free fatty acids (FAs), including unesterified FAs, are present in both the follicular fluid and the oocyte–cell complex (OCC) and are involved in the formation of the OCC follicle environment, which is directly related to oocyte quality [85].

In vitro studies have confirmed the positive or neutral effects of n-3 PUFA treatment on oocyte maturation and developmental competence [86]. Sharma et al. [76] reported that increased follicle size in cows improves both oocyte quality and corpus luteum (CL) function. In cattle, on the other hand, PUFA supplementation during in vitro studies has led to disruptions in the expression of genes involved in the lipid metabolism of hill cells. These changes modify the molecular mechanisms that regulate oocyte maturation [74]. Then, there are contradictory findings on the effect of n-3 PUFA on oocyte and embryo development [87], suggesting that feeding cows flaxseed has no effect, which showed a reduction in oocyte fertilization and embryo quality. A study by Moallem et al. [88] suggested a direct beneficial effect of n-3 PUFA fatty acids on the ovaries. In fact, in vitro embryo production performed after transvaginal ultrasound-guided oocyte retrieval (oocyte retrieval) without ovarian stimulation was increased after n-3 PUFA supplementation compared to a control consisting of saturated FA supplementation.

Dietary n-3 PUFA supplementation may be particularly recommended in cows with high genetic potential and unpredictable oocyte quality [89]. Studies on the effects of n-3 fatty acids (N-3 PUFAs) on reproduction in ruminants mainly focus on the study of biomarkers that regulate ovarian function, ovarian processes such as steroidogenesis, and oocyte competence [90].

The modification of fatty acids in animal diets can affect the concentration of circulating metabolic hormones and steroid hormones in the ovaries. This can affect the development and capacity of ovarian follicles and the composition and secretion of reproductive tissues. One of the parameters determining the reproductive success of animals is prostaglandin (PG) concentrations. This allows us to assess the effect of n-3 PUFAs on processes in the uterus that regulate the estrous cycle and pregnancy formation [91]. Prostaglandins (PG) play a key role as local signaling molecules. Their precursor is arachidonic acid (AA; C20:4), which is found in cell membranes as a component of the phospholipid bilayer [92]. The process of AA release from cell membranes occurs through the action of the enzyme phospholipase A2 (prostaglandin-endoperoxidase 2—PTGS2), previously known as cyclooxygenase, which converts AA into the prostaglandin PGH2 [93]. PGH2 is a precursor for various series 2 prostaglandins, such as PGF2α, PGE2, PGD2, PGI2, and thromboxane A2 (TXA2) [94].

**Figure 4:**
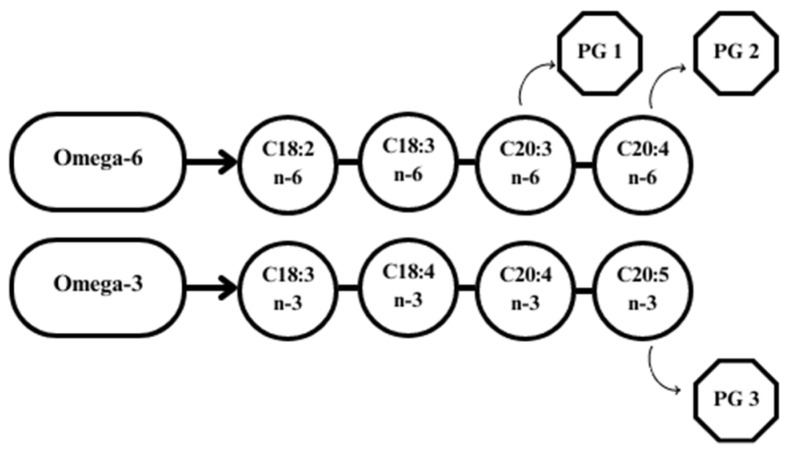
Metabolism of prostaglandins series 1, 2, and 3 (based on Park et al. [95]). Explanations: C18:2 n-6—linoleic acid; C18:3 n-6—γ-linolenic acid; C20:3 n-6—dihomo- γ linolenic acid; C20:4 n-6—arachidonic acid; C18:3 n-3—α-linolenic acid; C18:4 n-3—stearidonic acid; C20:4 n-3—eicosatetraenoic acid; C20:5 n-3—eicosapentaenoic acid; PG1, PG2, PG3—prostaglandin 1 or 2 or 3.

An increased proportion of n-6 fatty acid-rich components (e.g., corn, soybeans) in the ration has been shown to contribute to the synthesis of PG series 1 and 2, while n-3 supplementation induces the production of PG series 3 (Figure 4) [95].

During diestrus, there is an increase in the expression of the cyclooxygenase enzyme PTGS2, which leads to the synthesis and release of prostaglandin PGF2α, which is associated with the process of luteolysis or yellow body atrophy, which is crucial in regulating the estrous cycle [44,96]. In contrast, early in pregnancy, there is an increased production of prostaglandin PGE2 under the influence of type I interferon, which regulates the expression of the enzyme PTGS2 in the endometrium [97]. PGE2 helps maintain the luteal phase, which is essential for maintaining pregnancy and promotes embryo elongation and implantation. N-3 PUFA, especially docosahexaenoic acid (DHA), is a potent inhibitor of the activity of the enzyme PGHS (prostaglandin endonadoxide synthase) involved in the production of eicosanoids. Reducing the synthesis of PGE2s is a beneficial process as the excess of prostaglandin E-2s contributes to abnormalities in oocyte meiosis and fertilization [83]. In addition, a significant proportion of bovine embryos are believed to fade due to the insufficient inhibition of uterine PGF2α. There are several mechanisms contributing to the reduction in prostaglandin F2α activity. The first is a reduction in the expression of genes involved in uterine PG synthesis. A study by Dirandeh et al. [77] showed reduced PGFM concentrations in cows whose rations were based on elevated α-linolenic acid, indicating a significant reduction in the amount of PGF2α secreted in the uterus. Cows subjected to the test diet also had a reduced expression in PGFS, which converts prostaglandin H2 (PGH2)—an unstable intermediate compound in the PG biosynthesis pathway—into prostaglandin F2α. Research by Plewes et al. [98] illustrated another important effect of changing the fatty acid composition of membrane phospholipids on reducing PGF2α secretion. Cows in early pregnancy fed for 60 days on feed supplemented with n-3-rich fishmeal were characterized by, among other things, a modified membrane lipid microdomain, resulting in reduced prostaglandin F2α signaling. As the researchers explained, the mechanism of the PUFA’s effect on membrane lipid microdomains involves altering the distribution of protein molecules, and, subsequently, transforming the protein–lipid complex or protein–protein interactions. Moreover, n-3 family fatty acids can affect the availability of arachidonic acid (AA) in endometrial cells [99]. Studies have shown that the endometrium of lactating dairy cows contains a significant amount of AA acid on day 17 of the estrous cycle (about 15.3 g/100 g FA) when PGF 2α secretion is much lower than during the peripartum period [100]. N-3 fatty acids have been shown to effectively reduce AA concentrations in animals. Giller et al. [91], after supplementing the diets of heifers with 450 g of rumen-protected fish oil (omega 3 FA), observed a reduction in the concentration of AA in their bodies. This effect is a result of the specific metabolism of n-3, in which the process of competitive inhibition of Δ6 desaturase takes place. This is to prevent the synthesis of arachidonic acid (AA), excluding it from the phospholipid bilayer and competitively inhibiting the enzyme cyclooxygenase-2 (COX-2) [101].

The dietary supplementation of dairy cows with n-3 PUFA has been associated with a larger corpus luteum (CL) size and higher blood progesterone levels, which, in turn, increases the number or size of ovarian follicles [102]. Freret et al. [103] also reported that cows receiving a diet supplemented with flaxseed showed an increase in the size of pre-ovulatory follicles and small follicles. This relationship was confirmed by a study by Ulfina et al. [104] in which cows received either a supplement of crushed flaxseed at 750 g/day/head, 250 g/day/head of butyric acid, or a diet without additives. They found that cows given flaxseed, which is a source of n-3 fatty acids, had a significantly larger dominant ovarian follicle size (12.78 ± 0.62 vs. 10.54 ± 0.57) and larger corpus luteum size. In contrast, in a study where Sinedino et al. [105] used algae supplementation in the diets of dairy cows, at 0 or 100 g/cow of algae product containing 10% DHA, from day 27 to 147 postpartum, it was shown that supplementation had no effect on either ovarian follicle diameter or progesterone concentration. The effect of feeding hemp seed cake on reproductive parameters in cattle was studied by Windersen et al. [106]. Angus heifers whose diets contained 20% hemp cake as a substitute for 20% dried corn distillers’ broth were characterized by the regulated concentrations of the microorganisms present in the vagina and uterus. According to the authors, the achieved effect is important from the point of view of the reproductive parameters, since the bacterial infection of the uterus can contribute to disorders in the development of ovarian follicles. This is due to the secretion of lipopolysaccharides (LPSs) and tumor necrosis factor-α (TNF-α), which can limit ovarian follicle growth and estradiol production. As a result, the probability of maturation and ovulation of the first dominant ovarian follicle in infected cows is lower. The results obtained may be determined by the antimicrobial components contained in hemp cake, such as cannabinoid derivatives, which affect a set of Gram-positive and Gram-negative pathogens [107].

Reproductive performance in ruminants plays a key role in the profitability of animal husbandry. However, reproductive disorders in dairy cattle are major problems contributing to infertility and low dairy productivity. Insufficient or unbalanced nutrient intake can cause reproductive failure through the malfunction of reproductive organs [108]. Major metabolic changes are required to meet production demands. Dietary manipulation to improve fertility has shown great promise. A proper supply of n-3 polyunsaturated fatty acids (PUFA n-3) in the diet of dairy cows can increase the chances of conception [102] and reduce the risk of pregnancy loss [77]. Dietary supplements can be useful in improving the reproductive performance of cows by helping to reduce the negative energy balance that occurs after parturition, leading to an earlier return to the estrous cycle and improved fertility [99]. As a result, providing an adequate diet and supplementation can improve the reproductive performance of dairy cows, which is key to increasing milk production efficiency and sustainability in the livestock industry [109]. The results of the observed effect of n-3 FA supplementation on the reproductive performance in cows are summarized in Table 1.

The use of flaxseed in various forms in ruminant nutrition during the breeding season contributes to improved reproductive performance and better nutrient utilization, which determines measurable health benefits [110]. Swanepoel and Robinson [111] reported that the use of a flaxseed component characterized by a high content of alpha-lipoic acid (ALA) in the feeding of dairy cows has a significant effect on conception rates and reduces the number of abortions. A study conducted by Sinedino et al. [105] showed that supplementing the diet of dairy cows with seaweed rich in docosahexaenoic acid (DHA) at 100 g per cow, from 27 to 147 days postpartum, had a positive effect on animal reproduction. Increased levels of interferon (IFN)-stimulated genes in peripheral blood leukocytes (PBLs) were observed, indicating better reproductive fitness. In addition, more pregnancies were observed in algae-fed cows, both primiparous and multiparous, which is associated with enhanced embryonic development due to increased interferon-τ (IFN-τ) production. Daily dietary supplementation with DHA-rich algae improved estrous cyclicity and pregnancy rates at first artificial insemination in primiparous cows, as well as overall pregnancy rates, and reduced time to pregnancy in all cows. An examination of peripheral blood leukocytes from pregnant cows fed algae also showed increased expression of the RTP4 gene, which was associated with the amount of interferon-tau secreted by the fetus compared to the control group [105]. Elis et al. [102] analyzed the effects of n-3 fatty acids on the reproductive parameters of 46 Holstein cows. Animals that received a basal diet along with the addition of protected fish oil at 1% of dry matter between calving and 2 months after calving were characterized by a reduced rate of failure to fertilize and/or early embryo mortality. The study by Gonzalez et al. [112] was conducted on 112 animals: 61 heifers and 51 Holstein Friesian cows. The animals were randomly divided into two experimental groups. The first group consisted of 66 animals (30 cows and 36 heifers), which received a commercial concentrate with added fats from extruded flaxseed (source of linolenic acid) and soybean oil (source of linoleic acid). The second group, consisting of 46 animals (21 cows and 25 heifers), received no fat supplements but were given the same diet as the animals in the first group without fat supplements. The supplementation of the first group lasted from 3 weeks before calving until the second month of lactation. The results suggest that supplementation had a positive effect on conception rates in heifers, reducing the days from calving to the first artificial insemination. In contrast, no significant differences were observed between treatments in reproductive parameters in cows. Velazquez et al. [113] showed in their study that supplying n-3 PUFAs to the diet of small ruminants during pregnancy alters the physiology of the offspring. An analysis of the fetuses of animals fed 10 g/kg Ca salt (a source of n-3 PUFAs) from day 100 to 145 of gestation showed increased liver weight compared to the control group. In addition, beneficial effects on the development and growth of the offspring were observed, as well as the modification of the expression of immune mediators, enzymes involved in DNA methylation, and an acceleration in fetal growth.

**Table 1:** Impact of n-3 fatty acid supplementation on reproductive performance—research overview.

Experimental Animals	Experiment Design	Number of Fatty Acids in the Ration/Fat Supplement	The Observed Results	Source
739 primiparous and multiparous Holstein cows	Cows were assigned randomly to either a control or the same diet supplemented daily with 100 g/cow of an algae product containing 10% DHA Experiment duration: 27 to 147 days postpartum	Control diet: n-3—3.41% n-6—40.66% Algae supplement: n-3—30.50% n-6—6.56%	↑ Resumption of estrous cyclicity ↑ Pregnancy first in primiparous cows ↑ Number of pregnancy primiparous and multiparous cows ↑ Expression of RTP4 in peripheral blood leukocytes ↑ Increased the incorporation of DHA, EPA, conjugated linoleic acid isomers cis-9 trans-11, trans-10, cis-12, and total n-3 FA in plasma phospholipids	[105]
42,256-day pregnant Israeli Holstein dry cows	Cows were supplemented with encapsulated fats in treatments designated as an SFA—saturated fat at 240 and 560 g/day per cow, prepartum and postpartum (PP), respectively; FLX—flaxseed oil at 300 and 700 g/day per cow prepartum and PP, respectively; FO—fish oil at 300 and 700 g/day per cow prepartum and PP, respectively	FLX: SFA—62.41% MUFA—8.10% PUFA—26.49% n-3—23.42% FO: SFA—71.73% MUFA—16.66% PUFA—11.60% n-3—7.68%	FO: ↑ Proportion of docosahexaenoic acid (DHA) ↑ The follicle number during ovum pickup ↑ Percentage of oocytes that developed to blastocysts Oocyte cleavage FLX: ↑ The follicle number during ovum pickup ↑ The proportion of a-linolenic acid (ALA) in follicular fluid, granulosa cells, and cumulus–oocyte complexes ↑ Oocyte cleavage	[88]
37 Angus heifers (Bos taurus) aged between 10 and 27 months	Angus heifers were supplemented with either 450 g of rumen-protected fish oil (omega 3 FA) or sunflower oil (omega 6 FA). Experiment duration: 56 days	A diet with fish oil: SFA—72.8% MUFA—8.04% PUFA—19.1% n-3—13% n-6—5.7% n-6/n-3—0.46 A diet with fish oil: SFA—70.8% MUFA—10.1% PUFA—19% n-3—4.1% n-6—15.1% n-6/n-3—3.91%	A diet with fish oil: ↑ Embryo elongation ↑ Concentration of plasma progesterone during luteal growth ↑ Increased plasma P4 concentration ↓ Endometrial concentration—precursor of arachidonic acid	[91]
315 early lactation Holstein cows	Cows were offered rations formulated to contain 0 g/kg (No-Lin), 25 g/kg (LoLin), and 50 g/kg (HiLin) dry matter (DM) of LinPro—products based on flax seeds	NoLin diet: * SFA—24.76% MUFA—26.46% PUFA—48.47% n-3—4.42% LoLin diet: * SFA—22.85% MUFA—26.12% PUFA—50.73% n-3—10.23% HiLin: * SFA—21.47% MUFA—26.43% PUFA—51.88% n-3—15.53%	LoLin, HiLin diet: ↓ Fertilization frequency ↓ The reproductive performance ↓ The body condition score (BCS) in early lactation with LinPro feeding ↑ Increased plasma P4 concentration or HiLin cows ↑ The health status of cows	[111]
120 nonlactating pregnant Holstein cattle	Prepartum cattle were fed 1 of the following 3 diets: (1) no fat supplement (CON); (2) 1.15% of dietary DM as Ca-salts of soybean oil (CSO, 140 g/cow/daily) supplement; (3) 1.15% of dietary DM as Ca salts of fish oil (CFO, 140 g/cow/daily) supplement	CSO: SFA—20.4% UFA—76.8% PUFA—60% n-3—13.6% n-6—46.4% CFO: SFA—26.1% UFA—48.2% PUFA—51.2% n-3—14.7% n-6—31.1%	CFO: ↓ The period between first estrus and first insemination ↑ The health status of cows ↓ Total reproductive disorders ↑ Improved productive and reproductive performance in the subsequent lactation	[114]

* calculated based on the reported fatty acid profile; ↑ increase; ↓ decrease. Explanations: BCS—body condition score; CFO—Ca salts of fish oil; CON—no fat supplement; DM—dry matter; FLX—flaxseed oil; FO—fish oil; HiLin—diet with 50 g/kg of flax seeds; LoLin—diet with 25 g/kg of flax seeds; No-Lin—diet with 0 g/kg of flax seeds; P4—plasma 4; PP—postpartum; TP4—receptor Transporter Protein 4; SFA—saturated fatty acids; UFA—unsaturated fatty acids; MUFA—monounsaturated fatty acid; PUFA—polyunsaturated fatty acid; N-3—omega-3 fatty acids; N-6—omega-6 fatty acids; ALA—α-linolenic acid; DHA—docosahexaenoic acid; EPA—eicosapentaenoic acid.

## 5. Role of N-3 Fatty Acids in the Cows’ Nutrition during Periparturient Period

The supplementation of n-3 fatty acids during the transition period favorably affects the energy balance of cows by mitigating the effects of subclinical inflammation [115]. Recent studies indicate that systemic inflammation can negatively affect nutrient homeostatic mechanisms, which can lead to various adverse effects. Such effects include decreased feed intake, increased concentrations of non-esterified fatty acids (NEFAs), hyperketonemia, and an increased risk of disorders and diseases such as hepatic steatosis [116]. Lopreiato et al. [117] report that the expression of adhesion molecules involved in inflammatory interactions between leukocytes and endothelial cells is reduced by all n-3 PUFAs. According to a study by Greco et al. [44], reducing the ratio of n-6 to n-3 fatty acids in the diet of lactating dairy cows while maintaining similar overall fatty acid concentrations resulted in improved production performance during early lactation. In addition, providing more n-3 fatty acids and less n-6 fatty acids resulted in a reduced acute inflammatory response following the intramammary lipopolysaccharide (LPS) challenge. This nutritional strategy might be used to improve the metabolic status of cows during the transition period, which not only modifies immune cell function but also reduces the inflammatory response. An experiment conducted by Pi et al. [116] shows the effect of flaxseed oil and rubberseed oil supplementation on the immunological status of dairy cattle. The results show that enriching the diet with n-3 family fatty acids from flaxseed oil can also enhance immune function by reducing pro-inflammatory factors such as TNF-α and IFN-γ. TNF-α is an important mediator of the inflammatory response that regulates neutrophil recruitment and activation. In contrast, IFN-γ, as an important pro-inflammatory cytokine, is produced by T helper 1 (Th1) cells that initiate cellular immune responses. However, the study showed that IgG levels were significantly higher in the group receiving rubberseed oil alone compared to the group supplemented with flaxseed oil. This may be due to an increase in the (n-3)/(n-6) PUFA ratio in the serum of cows receiving the oils. The EFA and PUFA contents of the total rubberseed oil were 83% and 59%, respectively. Both flaxseed oil and rubberseed oil have high levels of ALA. In the former, ALA accounts for about 55% of the total FA content of the oil; meanwhile, in the latter, the ALA content is lower at 22% [116] Serum antioxidant activity is related to the activity of antioxidant enzymes and the content of lipid peroxides produced by free radicals or reactive oxygen species EFAs. Antioxidant enzymes such as SOD, GSH-Px, and CAT have been shown to have the ability to scavenge free radicals [118]. Due to the active hydrogen atom in PUFA, an increase in free radicals increases the possibility of spontaneous oxidation [117]. In a study by Pi et al. (2019) [116], there was a significant increase in serum PUFA (CLA and ALA) levels, accompanied by a significant decrease in GSH-Px and CAT activity and an increase in lipid peroxide (MDA) levels in the groups receiving the added oils compared to the control group. Moreover, the serum PUFA (ALA and cis -9, trans -11 CLA) content was positively correlated with MDA concentrations and negatively correlated with serum GSH-Px concentrations, indicating that an increase in PUFA levels may contribute to a decrease in antioxidant capacity and an increase in lipid peroxidation products. The addition of flaxseed in animal feed rations may also help reduce the negative health effects associated with oxidative stress and stimulate the body’s antioxidant defense system [119]. In dairy cows, feeding experiments have shown that flaxseed has antioxidant properties [120,121]. This effect is primarily determined by the high content of lignans, which are a source of natural antioxidants [122]. In addition, they are among the polyphenolic compounds that exhibit multidirectional effects on animal organisms [123]. In a study by Gandra et al. [124], the effect of using flaxseed at 60 and 80 g/kg and soybean seeds at 120 and 160 g/kg of ration on the immune system of dairy cows in transition was demonstrated. It was shown that the type and timing of supplementation had a significant effect on the percentage of circulating leukocytes, monocytes, and neutrophils, showing positive phagocytosis in both the prepartum and postpartum periods. Research diets containing additional sources of n-3 fatty acids (flaxseed) helped increase the overall phagocytosis capacity of leukocytes (WBC) by 69% and monocytes (MONO) by 97.5%. Flaxseed is characterized by a high content of n-3 acids. In their chemical composition, that with the highest concentration is α-linolenic acid (C18:3), which is a structural material for the cell membranes of animal tissues and is a specific initiator of the synthesis of paracrine hormones that regulate physiological processes, among others, such as prostaglandins. The products formed during the metabolism of α-linolenic acid are characterized by anti-allergic, anti-carcinogenic, and beneficial effects on the circulatory system [125]. In the case of n-6 acids, the immune efficiency of WBC increased by 56.7% and MONO by 87.7%. Considering the results of this study, it can be concluded that the presence of the polyunsaturated fatty acids developed acquired an innate cellular immune phagocytosis due to an increase in monocyte efficiency and an increase in the expression level of adhesion molecules in lymphocytes, resulting in a pro-inflammatory response. The study observed an additional effect of fatty acids on the adaptive immune response, which is triggered when innate immune mechanisms are unable to eliminate a pathogen. It is characterized by the production of lymphocytes and memory cells that are antigen-specific and have the ability to recognize specific antigenic determinants of the pathogen. The results showed an increased percentage of helper T cells, cytotoxic T cells, and cells expressing IL-2 receptors and CD62 adhesion molecules. Sun et al. [55], in their study, compared the effects of a diet supplemented with n-3 and n-6 fatty acids on, among other things, immunological indices in the blood of cows and calves in transition. Animals (*n* = 45) at day 240 of gestation were divided into a control group and two research groups receiving 3.5% of extruded flaxseed as a source of PUFA n-3 and 8% of extruded soybeans as a PUFA n-6 supplement in the ration. The results showed that the diet of the cows containing n-3 fatty acids contributed to an increase in the concentration and activity of neutrophil granulocytes during the transition period. In addition, the pro-inflammatory interleukin IL-1β of maternal cows at transition and the cytokine TNF were shown to decrease in newborn calves. According to the authors, the anti-inflammatory activity of n-3 fatty acids may be related to the fusion of nuclear factor kappa B (NFκB), limiting the production of inflammatory cytokines. A study by Kra et al. [126] aimed to investigate the effects of n-3 fatty acids from flaxseed (FLX) or fish oils (FOs) on the proteome of peripheral blood mononuclear cells (PBMCs) in periparturient dairy cows. Group one received a basal diet supplemented with encapsulated fat providing ALA derived from flaxseed at a dose of 56.1 g/day/cow before parturition and 131.0 g/day/cow after parturition. In contrast, the diet of group two was supplemented with encapsulated fat derived from fish oil, providing EPA at 5.8 g/day/cow and DHA at 4.3 g/day/cow before delivery, as well as EPA at 13.5 g/day/cow and DHA at 10.0 g/day after delivery. The study quantitatively examined more than 3800 proteins to better understand the mechanisms of action of these fatty acids on the immune system of cows after parturition. In cows that received supplementation with n-3 fatty acids from flaxseed, an enrichment of the acute phase signaling system and complement system was observed, as well as a higher amount of RELA protein in PBMCs compared to the control group and the group receiving fish oils. The effect of n-3 fatty acid supplementation on other immune parameters was moderate. Peripheral blood mononuclear cells (PBMCs) are included among the body’s immune cells. They include lymphocytes and monocytes (T lymphocytes, B lymphocytes, NK cells, and dendritic cells). In healthy animals, they circulate in a resting state and monitor potential immune-relevant events; however, when necessary, they can respond quickly and effectively in an inflammatory manner [127]. The use of proteomics can reveal new proteins and enriched pathways in tissues or cells when animals are subjected to a specific factor, such as nutritional modification, that affects their physiology at the molecular level [128]. On the other hand, Bragaglio et al. [129] evaluated the effect of the supplementation of a diet rich in n-3 fatty acids supplemented with algae (*Schizochytrium* sp.) on cow immunity. Twenty-one multiparous Italian Friesian cows at 220 ± 20 days of lactation were evenly divided into three groups: a control group that received no supplementation, an experimental group (D) receiving a supplement of 136 g of docosahexaenoic acid (DHA) daily, and a group (E) supplemented with 136 g of DHA + 2000 U.I. vitamin E daily. All animals received a control diet for the first 2 weeks from the third to the sixth week, and the feeding of the experimental groups was supplemented with acids of the n-3 family from algae, which are distinguished by their significant content of long-chain polyunsaturated fatty acids (LC-PUFAs), such as docosahexaenoic acid (DHA) and eicosapentaenoic acid (EPA). They are considered a sustainable source of these essential fatty acids (EFAs). In addition to EFAs, microalgae also contain high levels of proteins (28 to 71%), lipids (10–20%), and carbohydrates (5–15%), as well as a variety of minerals and pigments. They also contain antioxidant substances that have the potential to protect against oxidative stress and damage to animal cells [125]. The results of a study by Bragaglio et al. [129] showed a significant interaction between time and the experimental group for the activation of the humoral immune response. Both groups that received supplementation showed an increased antibody response at weeks 4, 5, 6, 7, and 8 after antigen administration, indicating the action of a secondary immune response; meanwhile, no differences were observed in the primary response at weeks 1, 2, and 3. Dietary supplementation with seaweed during the late lactation period had a beneficial effect on stimulating the cattle’s immune system, enhancing both cellular and humoral immune responses.

## 6. Importance of FA N-3 in Fetus Developmental Programming and Embryo Development

Proper fetal growth is essential and critical to an individual’s long-term performance and health [130]. The intrauterine environment plays an important role in fetal development. Fetal programming theory states that environmental stimuli, including nutrition during the fetal development stage, have effects of a long-term nature. N-3 polyunsaturated fatty acids (PUFAs) are essential for normal physiological function and animal health. The level of these acids in the mother’s blood can affect fetal growth and development [74]. During pregnancy, dairy cows have been shown to benefit from the supplementation of polyunsaturated fatty acids, especially n-3 fatty acids [113,114]. In addition to the effects of fatty acids on cell membrane fluidity and biological properties, they show positive effects on many aspects of dairy cattle reproduction [72]. They contribute to the improved development of the ovarian follicle [82], the oocyte [131], and the embryo [132]. Additionally, they increase the expression of genes involved in reproductive processes [74]. The effects of n-3 fatty acid supplementation are particularly promising. Research by Roque-Jiménez et al. [73] showed that they have a positive effect on imprinting during gametogenesis. The results of Freret et al. [103] showed the beneficial effects of these acids on embryonic development, and research by Elis et al. [102] suggested their positive effects on the developing fetus, which requires significant amounts of fatty acids to support rapid cell growth and activity, and, among these, n-3 and n-6 polyunsaturated fatty acids (PUFAs) are key [133]. Therefore, maternal nutrition during pregnancy plays a key role in the performance of adult offspring [134]. In addition, supplementation with n-3 fatty acids can have beneficial effects on development and function after birth, as demonstrated in a study by Opgenorth et al. [135], which linked long-term health consequences in various animal species to adverse prenatal (in utero) gastrointestinal exposure [136].

During the prenatal stages, fatty acids and their metabolites play a key role in the processes of cell growth, differentiation, and regulatory response between metabolic and neuroendocrine environments [137]. An important feature that regulates embryonic development is likely to be lipid absorption and metabolism [128]. Insufficient, unbalanced, or excessive fatty acid intake in the early stages of development may contribute to both metabolic and nutritional programming [73]. Prior to embryo implantation in ruminants, the transformation of the uterine epithelium for trophoblast attachment and implantation requires specific changes in the transcriptome [138]. The trophoblast is the outer layer of cells that is formed early in embryonic development in mammals [139]. In this process, progesterone (P4) plays a key role as it is essential for the proper development of the endometrium, which is necessary for the proper development of the fetus, its implantation, and its growth until delivery [140]. The production of progesterone in the uterus creates a friendly intrauterine environment that promotes the embryo in its development and is crucial for the proper formation of the extraembryonic membranes. Fat sources rich in n-3 fatty acids act as cyclooxygenase inhibitors in endometrial tissue [90]. As a result, PGF2α secretion in the endometrium may be suppressed, potentially counteracting early embryonic death by preventing the regression of the corpus luteum in the ovary and allowing continued progesterone production, which, in turn, promotes embryonic survival [141]. Leroy et al. [72] explained this fact by saying that n-3 fatty acid supplementation during the periconceptual period can weaken the inflammatory force, leading to a greater chance of embryo survival. In contrast, Carneiro et al. [142] claimed that exogenous supplementation can minimize the percentage of bovine embryos that are lost due to the insufficient inhibition of PGF2α secretion while increasing embryo survival rates.

In a study conducted by Kumar et al. [110], heifers were supplemented with soybean oil (n-6 source) or flaxseed oil (n-3 source) at a 3.5% dose. Animals receiving the n-3 source were distinguished by increased progesterone levels (by 82.6%). In addition, higher levels of other hormones of reproductive importance such as insulin by 13.1% and IGF-1 by 20.9% were observed. In cattle, just before implantation, the trophoblast undergoes rapid elongation, which begins between days 7 and 13 of gestation. In response to ovarian P4, major changes occur that are necessary to elongate the embryo and prepare the uterus for implantation, regardless of whether a properly developed embryo/conception is present [143]. These changes are crucial to the proper course of pregnancy and the success of embryo implantation. At this stage of development, mononuclear trophoblast cells synthesize large amounts of interferon-tau (IFN-τ), which is a signal of pregnancy recognition by the maternal body [144]. IFNTs trigger the activation of genes associated with the interferon response in the endometrium [145]. These genes, known as interferon-induced genes (ISGs), affect endometrial and trophoblast function to promote embryo reception and implantation [146]. Progesterone and interferon-τ work together to ensure proper endometrial health and pregnancy maintenance by inhibiting luteolysis and initiating signals that enable the maternal body to recognize pregnancy. These complex processes are crucial to the proper course of pregnancy and the success of embryo implantation [132]. Matras et al. [147] reported that supplementing dietary intake with n-3 fatty acids, especially EPA and DHA, which block prostaglandin PGF2α synthesis, supports the action of interferon τ, reducing the risk of preimplantation embryo death. It has been shown that n-3 acids can act together with embryo-derived interferon-τ to prevent the onset of luteolysis and facilitate pregnancy by inhibiting progesterone release [89]. A study by Giller et al. [91] on the use of 450 g of rumen-protected fish oil (n-3 FA) in heifer diets showed increased interferon-tau activity. In addition, the process of embryonic elongation was increased in these animals compared to the group receiving sunflower oil (n-6 FA). In vitro results also suggest an improvement in blastocyst rates, which were observed especially in low-quality oocytes under the influence of supplementing cows with n-3 fatty acids [74]. In addition, studies have shown that in vitro maturation medium (IVM) supplemented with a low dose of DHA also contributed to increased rates of embryo development [74]. Another study by Azam et al. [148] also confirmed the beneficial effects of n-3 PUFA on embryonic development in the bovine embryo. The supplementation of 100 μm ALA to IVM medium increased the number of morulae and also improved the subsequent early embryonic development of the embryo.

In particular, polyunsaturated fatty acids (PUFAs), such as linoleic acid (LA) and alpha-linolenic acid (ALA), as well as their metabolites, such as docosahexaenoic acid (DHA, 22:6n-3) and arachidonic acid (AA, 20:4n-6), are transported across the placenta to the developing fetus [149]. Because of their key role in cognitive development, long-chain FA derived from LA and ALA are particularly important for the developing fetus [150]. Embryonic growth is mainly dictated by the availability of these nutrients in the maternal circulation and the ability of the placenta to transport them into the fetal circulation [151]. They have a significant impact on the development of the central nervous system (CNS) in the fetus [152]. DHA and EPA fatty acids play a key role in fetal brain development, the formation of certain elements of the nervous system, retinal maturation, and influence neonatal behavior [73]. Maternal fatty acids (FAs) pass through the placenta and accumulate in various fetal tissues. The passage of these substances across placental gradients depends on the availability and activity of specific transporters, including fatty acid transporter proteins (FATPs), fatty acid translocase (FAT/CD36), and intracellular FA-binding proteins (FABPs) [153]. Changes in mRNA expression in the fetal placenta in response to n-3 PUFA supplementation have been noted. One of the genes affected by this supplementation was the DNA methyltransferase (DNMT)-3A gene, which is responsible for DNA methylation. In addition, the free fatty acid receptor (FFAR)-4, which plays an important role in lipid metabolism, was transformed under the influence of PUFA n-3 [73]. In ruminant animals, it has been observed that the transfer of some unsaturated fatty acids appears to be restricted in the epithelial-vascular placenta, often resulting in a deficiency of these components in newborns [130]. In a study conducted by Desantadina et al. [154] on the placenta of cattle, changes in mRNA expression were noted only for the fatty acid transport protein FATP-1. In early pregnancy, FATP-1 shows a higher expression on the fetal side compared to the maternal side. However, in the second half of pregnancy, this difference decreases, and, at the end of the pregnancy, mRNA concentrations on both sides of the placenta become similar. This indicates that FATP-1 plays an important role in fatty acid transport early in fetal development; however, later, its role seems to diminish. Interestingly, in calves, fatty acid transfer through colostrum or milk appears to be more important than transfer across the placenta [155]. Salehi et al. [156] showed that feeding colostrum and transition milk from mothers fed EFAs improved EFA availability to young calves. The ratio of LA to ALA affects the availability of EFA metabolites, as the two types of EFAs compete with each other for enzymes involved in their synthesis [157]. In addition, the combination of EPA and DHA regulates placental lipid metabolism and storage by affecting the esterification of placental n-3 EFA lipids [158]. Various functions of the placenta, such as angiogenesis, inflammation, and oxidative stress, are regulated by an adequate intake of n-3 fatty acids. All of these aspects underscore the importance of the proper delivery of maternal LCPUFAs to the fetus, in which placental transport plays a key role in providing essential nutrients [159].

## 7. N-3 Fatty Acids Are a Key Component in Building Immunity 

The Intensification of livestock production is fraught with several factors that have the effect of reducing the immune status of animal organisms, which often has a measurable impact on animal health [160]. The overriding elements that determine abnormalities in the functioning of the immune system are genetically determined factors or arise as a result of the exposure of the animal to various types of immunosuppressive agents [154]. Some of the main etiological factors determining immune deficits are multiple strains of pathogenic bacteria, viruses, fungi, and protozoa [161]. Moreover, inadequate management methods and qualitative and quantitative deficiencies in the composition of the feed ration [162], oxidative stress [163], and lack of welfare [164] contribute to the occurrence of diseases caused by abnormalities in major physiological processes [165,166]. A common consequence of an animal’s exposure to the aforementioned factors is the occurrence of cumulative stress, which lowers the humoral response of the immune system, disrupting the homeostasis of the animal’s body. The perinatal period is considered a critical stage in the life of high-yielding dairy cows and is characterized by sudden physiological changes that require rapid adaptation [167,168]. During this time, the cow’s immune system experiences many changes that can affect her immunocompetence [169]. Reduced immunity is observed in late lactating cows, and available data suggest that the immune system may be impaired during the perinatal period. Despite the low incidence of diseases (metabolic and infectious) before calving, many reports indicate metabolic and immune differences during the drying-out period [161]. Jahan et al. [168] explained this disorder as changes in immune mechanisms and the innate immunity of cows despite an increase in leukocyte levels. This leads to an overall impairment of immune capacity, which contributes to immune dysfunction and is associated with a higher incidence of infectious diseases. A properly functioning immune system should protect dairy cows from various pathogens, including viruses, bacteria, and parasites [170]. The resulting changes in cattle immune mechanisms are a major contributor to the severity of health disorders [171]. When cows in transition are unable to adapt physiologically to the increased nutrient requirements associated with fetal growth and milk production, metabolic stress occurs, resulting in excessive lipid mobilization, oxidative stress, and inflammatory dysfunction. This also indicates a negative impact on the immune function, health, and production of dairy cattle, potentially affecting the immune capacity and development of the newborn [46,172,173].

The nutritional status of dairy cows and the metabolism of specific nutrients also influence the performance of the immune system [162]. Some mediators of the immune system can have reciprocal effects on the metabolism of nutrients, including fatty acids. Using various animal models as an example, it has been shown that their high nutritional availability contributes to improving their lymphocytic response to the presence of pathogens, regulating the activity of specific immune cells’ natural killer (NK), and reducing the number of interleukins (IL)-1β and IL-6, which are mediators of the immune response [174]. Therefore, any disturbance in nutritional or immune homeostasis can cause harmful feedback loops that further exacerbate health disorders and increase production losses [163]. In the immune system, mononuclear cells, such as the monocytes and macrophages, play a central role. Monocytes are derived from myeloid precursor cells in major lymphoid organs, such as the bone marrow and fetal liver, during both embryonic and adult hematopoiesis [175]. To protect the animal organism, the immune system uses a complex and dynamic network of lymphoid organs, cells, and humoral factors, which are divided into two distinct categories—innate immunity and acquired immunity—that exhibit varying rates and specificities in their respective response [161]. Innate immunity is the first line of defense against pathogen invasion, demonstrating a wide range of action and immediate, non-specific host defense to any tissue damage while also neutralizing potential pathogens. Depending on the effectiveness of the innate defense mechanisms, microorganisms can be eliminated within minutes to hours after invasion. Acquired immunity, on the other hand, is delayed compared to innate immunity and can take several days to mount a response to a specific threat. This is a more individualized and specific response to infectious pathogens, which can be enhanced by repeated exposure to the same microorganism [163,176,177].

Recent research by Bordon [178] has indicated that innate immune cells may also exhibit the ability to retain immune memory. This discovery alters the current understanding of many functions of the immune system. The innate and acquired immune systems must work synergistically to provide optimal protection against external threats and contribute to maintaining the optimal health status of cows [179]. Sordillo [163] has shown the important involvement of long-chain fatty acids (FAs) in the body’s immune response. Fatty acids both directly and indirectly affect the functions of the immune system as they play an important role in immune system processes, notably, the inhibition of arachidonic acid metabolism, induction of anti-inflammatory mediator synthesis, modification of intracellular lipids, and activation of nuclear receptors [124]. A study conducted by Radzikowska et al. [180] aimed to determine the effect of polyunsaturated fatty acid (PUFA) supplementation on the expression of immune regulatory genes in different cell types. The immunomodulatory effect of PUFAs was shown to depend on their chemical structure (e.g., n-3, n-6) and carbon chain length, as well as the specific cell type they act on, such as macrophages, neutrophils, epithelial cells, dendritic cells, innate lymphoid cells, T lymphocytes, and B lymphocytes. These diverse results indicate a comprehensive effect of PUFAs on the immune system, which may have important benefits for the health and functioning of the body. On the other hand, reports of the presence of n-3 FA in the diet, due to their chain length and the presence of double bonds at the carbon, show the strongest modulation of immune pathways compared to other fatty acids [181]. N-3 fatty acids and their metabolites can affect the function of multinucleated cells. Cell migration, phagocytosis capacity, the production of reactive oxygen species, and cytokines in neutrophil function are altered [182]. In the case of high exposure of the body to antigens, appropriate levels of ALA, EPA, and DHA in the diet can affect the immune response of macrophages through their polarization, that is, their activation and specialization in response to various immune signals [142]. In addition, ALA has a protective function by downregulating the expression of pro-inflammatory genes, which prevents a significant increase in inflammation and facilitates the migration of leukocytes from the blood to infected tissues upon the detection of bacteria by local cell populations. Then, n-3 supplementation prevents a significant increase in inflammation [183]. There are many published reports on the effects of n-3 fatty acids on the immune response of dairy cows. Olmo et al. [184] showed that long-chain n-3 PUFAs may be one strategy to enhance the innate immune function of dairy cows. Therefore, they are an important addition to cattle nutrition aimed at supporting the immune status of animals [126].

## 8. N-3 Fatty Acids as Immune Support for Newborn Calves

N-3 fatty acids also play an important role in supporting the immune and metabolic functions of dairy cattle offspring, as cows’ exposure to heat stress and limited or excessive energy intake during late gestation have been shown to affect their health status [46]. During the prenatal period, the placenta is an anatomical barrier separating the offspring from the mother’s body [185]. In newborn calves, the immune system in the first weeks of life is not yet fully developed. During this period, due to the often inadequate transfer of colostrum, resulting in a lack of passive immunity, calves are at risk of high morbidity and mortality [186]. In addition, the increased stress levels associated with the birthing process itself contribute to an increased production of reactive oxygen species that cause oxidative stress to the newborn [183]. The most common problems are gastrointestinal and respiratory diseases. Hence, optimal conditions during the initial rearing period determine the subsequent functional value and productive efficiency of calves. A key strategy for young animals is maternal nutrition during the final period of gestation and supplementation with supplements that are characterized by high bioactivity, determining, among other things, the provision of high-quality colostrum, which contains not only immunoglobulins, immunomodulatory factors, and antioxidant substances, but also long-chain fatty acids, which can play a key role in immune regulation and affect the oxidative status of newborn calves [46].

The protein fraction of colostrum contains immunomodulatory components such as immunoglobulins, lactoferrin (LF), and lysozyme (Lz), as well as the albumins β-lactoglobulin (β-LG) and α-lactalbumin (α-LG). Immunoglobulins (Igs) are bioactive immune proteins that play an important role in regulating the body’s humoral response. Their functions include the activation of the complement system, phagocytosis, and the ability to bind antigens. Various classes of immunoglobulins are found in colostrum, including IgG, IgA, IgM, and IgE. The ingestion of fat contained in colostrum further enhances thermoregulatory capacity [187]. The concentration of specific antibody classes in colostrum and milk is related to the current needs of the growing animal [188]. Due to Ig permeability in the intestinal mucosa of ruminants, passive immunoglobulin (Ig) transfer through colostrum occurs during the first 18 h of life. Increased Ig concentrations in the colostrum are associated with greater immune capacity transmitted on the body [160]. The colostrum immunoglobulins reflect the antigenic stimulation of the maternal system and are the most important parameter determining the success of passive immune transfer. Their adequate concentration in the colostrum ensures that animals establish their own immune defense mechanism and antioxidant system [189].

A study conducted by Wilm et al. [190] showed that serum IgG concentrations in dairy calves that achieved passive immune transfer decreased during the neonatal period (from 24 to 10 days of age) at a rate of about 0.7 mg/mL per day. In contrast, fatty acids in the colostrum and milk play a key role in mobilizing and regulating the immune system. Particularly valuable are unsaturated fatty acids (PUFAs), which exhibit antioxidant properties and reduce the activity of pro-inflammatory mediators [46]. A study by Moallem and Zachut [88] showed that feeding n-3 fatty acids to cows before calving can slightly increase the concentration of n-3 fatty acids in the blood of calves. The difference in the plasma fatty acid (FA) profile between newborn calves and their mothers was likely due to the low permeability of the bovine placenta to polyunsaturated fatty acids. Enrichment of the diet of dairy cows in late pregnancy with n-3 fatty acids increased the proportion of docosahexaenoic acid (DHA) but not α-linolenic acid (ALA) in the plasma of newborn calves. This was due to the necessity of DHA for proper fetal development. Therefore, n-3 FA supplementation in the colostrum can be considered as a sensible approach. This was confirmed in a study by Opgenorth et al. [135], where it was found that supplementation with n-3 fatty acids given with colostrum improved the oxidative status of newborn calves. In their study, calves given 60 mL of a 1:1 mixture of fish oil and flaxseed oil along with 200 mg of α-tocopherol in colostrum decreased the oxidative status index (OSi) in the first week of life. The results showed an increase in n-3 fatty acids and the presence of metabolites in plasma. The supplementation of fat-enriched colostrum contributed to a decrease in biomarkers of oxidative stress, indicating less free radical stress in supplemented calves but no change in oxidative status. It was concluded that the addition of n-3 fatty acids in the colostrum could have a significant effect on enhancing the anti-inflammatory status of calves. A study by Grodkowska et al. [191] also demonstrated the effect of supplementation with a mixture of fish oil and flaxseed on the level of immunomodulatory components in the colostrum of multiparous cows. The study involved 20 multiparous cows, which were divided into two experimental groups three weeks before the expected calving date: a research group, receiving a diet enriched with 150 g of fish oil and 250 g of flaxseed (*n* = 10), and a control group (*n* = 10). Colostrum samples for the study were taken twice a day on the first and second days of lactation, and, from the third to the fifth day of lactation, they were taken once per day. As a result of the experiment, it was found that supplementation with a mixture of fish oil and flaxseed significantly affected the basic chemical composition of colostrum. The concentrations of protein, fat, and casein were higher in the group receiving the fat supplement compared to the control group in all samples analyzed, except for the first sample taken at the initial stage of the experiment, where the casein content was higher in the control group. Colostrum from supplemented cows was characterized by a significantly higher protein concentration (by 1.94%). The results of the experiment showed that supplementation with a mixture of fish oil and flaxseed significantly affected the level of immunoglobulins in the cows’ colostrum. IgG, IgA, and IgM concentrations were higher in the experimental cows, with a significant increase in IgA and IgM concentrations. The content of lactoferrin in the colostrum of cows in the supplemented group was significantly higher compared to the control group, indicating a positive effect of supplementation on the level of this immunomodulatory protein. In addition, the results showed that supplementation increased the content of vitamin A in the colostrum of cows in the experimental group. Vitamin A has a significant impact on the body’s immunity; therefore, its higher content in colostrum may contribute to beneficial health effects in offspring.

Considering the results of the fatty acid analysis in colostrum, supplementation increased the content of DHA and C18:2 cis9 trans11-CLA fatty acids. The high content of docosahexaenoic acid (DHA) plays an important role in the development and function of the immune system [192]. Moreover, supplementation affected the favorable ratio of n-3:n-6 fatty acids in colostrum from the supplemented group, indicating a positive effect on the balance between these acids and potential health benefits in the offspring of supplemented cows. The results of the experiment indicate a significant effect of supplementation with a mixture of fish oil and flaxseed on the chemical composition of colostrum, especially in the context of immunomodulatory components, which may contribute to improving the health and immunity of cow offspring. A study by Santos et al. [120] evaluated the effects of açai oil fed to Holstein cows during the drying-out period on colostrum quality and the immune and antioxidant responses of their calves. Sixteen multiparous cows were divided into a control and a test group, in which the feed ration contained 4.48% of the analyzed raw material. The results of the experiment showed that the colostrum of cows fed with açai oil had a higher antioxidant capacity against superoxide radicals. Compared to the control group, an increase in IgG immunoglobulins by 40.4% was also observed, as well as an increase in IgA by 40%. In addition, a reduced lipoperoxidation index was observed, resulting in the increased uptake of immune cells in a calf’s intestine. The resulting immune effect of colostrum may be conditioned by its high constituent value of essential fatty acids (EFAs). On the other hand, the increase in its antioxidant capacity is due to the high concentration of antioxidants such as flavonoids, anthocyanins, and proanthocyanidins present in acai oil, among others. Calves born to cows fed açai oil tended to have higher serum total protein levels compared to calves born to control cows. This is due to increased levels of IgG- and IgA-class immunoglobulins in the colostrum given to the young animals.

The positive effect of n-3 fatty acids on the direct or indirect stimulation of immunity in newborn calves has been demonstrated in studies [155,193,194]. These studies also demonstrated the effect of fatty acids on the overall health status of calves. Dietary supplements, especially those rich in PUFAs, affect not only weight gain but also immune response, antioxidant status, and overall metabolism [155,195,196]. Lipids and proteins in foods for newborn animals are important sources of energy and fatty acids that have important structural and metabolic functions [197]. The composition of a milk replacer (MR) varies considerably depending on the type of raw materials used, which can come from milk, plants, or animals [198]. Several studies have shown that the use of different fats in milk replacer (MR) mixes or starters as feed for weaned calves can promote the development of immune responses and improve health [199] (Table 2).

A study conducted by El-Hamd et al. [202] on Holstein Friesian calves, with an average weight of 31.42 kg, showed a positive impact of flaxseed oil on newborn calves’ health status. The first group (G1; *n* = 14) served as the control group, and the second group (G2; *n* = 14) was supplemented with 0.2 mL of flaxseed oil/kg body weight from birth to weaning. The supplementation of the milk replacer with flaxseed oil significantly (*p* < 0.01) increased the number of red and white blood cells, percentage of monocytes, and neutrophils, as well as increased the plasma concentrations of the total protein, albumin, and globulin and improved the plasma immunoglobulin concentrations (*p* < 0.05) during the suckling period. Plasma glucose concentrations and AST and ALT activities were similar in both groups. Flaxseed oil typically makes up 32 to 45% of the seed weight. It is characterized by a high percentage of polyunsaturated fatty acids, in the composition of which α-linolenic acid (ALA; n-3) prevails, accounting for 55–57%, and the share of linoleic acid (LA; n-6) ranges from 15 to 18% [203,204]. It can be concluded that the enrichment of the calves’ diets with flaxseed oil contributes to the stimulation of the immune system of young cattle without negative effects on the hematological and biochemical parameters of the blood.

In a study conducted by Spitalniak-Bajerska et al. [205] on twenty-seven calves, a nutritional supplement to milk replacer in the form of ethyl esters of flaxseed oil (10 g/d) with freeze-dried apples or flaxseed oil (10 g/d) with freeze-dried apples was used. Supplementation with the flaxseed oil in the form of ethyl ester had a positive effect on health status, average daily weight gain, growth rate, and feed utilization. However, starter feed intake was lower in calves receiving the flaxseed oil preparation. Moreover, the addition of flaxseed oil ethyl esters reduced serum concentrations of triglycerides, total cholesterol, and LDL fractions and lowered total antioxidant capacity (TAS) and glutathione peroxidase (GPx) levels, which are responsible for protecting cells from oxidation through peroxides formed during biochemical processes. In addition, it led to a reduction in the levels of the circulating tumor necrosis factor (TNF). Flaxseed oil increased the serum levels of TAS and GPx. This confirms that supplementation with n-3 fatty acids improves oxidative function in young calves. Apples, used in this study as a carrier for fat supplements, are a source of antioxidants and are high in polyphenols, and this has a beneficial effect on FA stability [206]. In order to understand how n-3 FA supplementation can contribute to a reduction in oxidative stress, Opgenorth et al. [135] determined the effect of the supplementation of fish oil and flaxseed on polyunsaturated fatty acid (FA) and FA metabolite concentrations in plasma as inflammatory mediators and on the oxidative stress indices during the critical first week of calves’ lives. Twenty-four Holstein calves were randomly assigned to four experimental groups and received different doses of fish oil and flaxseed oil (0, 30, 60, or 120 mL) in a 1:1 ratio in the colostrum. All calves received 2.8 L of previously frozen colostrum that had ≥ 50 g/L of immunoglobulins. Supplementation with a mixture of fish and flaxseed oils added to colostrum had no effect on the health or growth of the calves. However, it decreased the phospholipid profile of n-6 FA: n-3 FA, increased the concentration of free and phospholipid n-3 FA, and increased several oxylipids derived from n-3 FA in the first week of life. Oxylipids are cell signaling molecules capable of mediating inflammation and its elimination through various mechanisms. The oxylipids that increased in the study are primarily the end products of EPA and DHA metabolism. 14,15-DiHETE, 17,18-DiHETE, and 5,6-DiHETE from EPA and 19,20-DiHDPA from DHA increased linearly with increasing n-3 FA supplementation. Of the aforementioned oxylipids, all are end products of cytochrome P450 enzymatic activity. Another oxylipid whose concentration increased with n-3 FA supplementation in calves is LXA 4. As reported by Kuhn et al. [207], LXA 4 is preferentially produced in cows during the perinatal period, most likely as a mechanism to reduce the systemic inflammation observed after parturition. Unlike oxylipids, isoprostanes are direct biomarkers of oxidative stress, as their production results from peroxidation induced by reactive oxygen and nitrogen species (RONS) and damage to the phospholipid membrane and, thus, cellular components [208]. Although the oxidative status index was not altered in the study by Opgenorth et al. [135], the administration of fish oil and flaxseed oil supplements reduced oxidative stress due to a decrease in plasma n-6 FA-derived isoprostane, 8-iso-PGA2, indicating a reduction in oxidative stress in calves during the first week of life. Karcher et al. [196] studied the effect of supplementing calves with fish oil or flaxseed oil on cytokine gene expression and growth rate. Forty-eight Holstein calves received a milk replacer (MR) alone (pork fat as a source of fat) or formula with 2% flaxseed oil or 2% fish oil supplementation. The study showed that flaxseed oil supplementation improved feed conversion rates compared to fish oil supplementation. However, the difference was not significant. Moreover, compared to the control group, none of the additives affected this index. Flaxseed oil supplementation induced a milder febrile response after provocation with the Pasteurella vaccine and reduced the expression of IL-4, IL-8, and osteopontin (Opn) in blood cells after in vitro LPS stimulation. These cytokines are particularly important as an element involved in controlling the inflammatory response, which may affect a young body’s ability to fight off disease. Decreased IL-4 expression may result from a weaker immune response to vaccination, leading to a reduced memory response of the adaptive immune system.

## 9. Conclusions

The presented results clearly confirm the importance and beneficial effect of n-3 family fatty acids on animal organisms, in this case, ruminants. The review focused mainly on two crucial aspects of cattle breeding: the reproduction of dairy cows and the health status and body condition of cows during the periparturient period, as well as the immunity of newborn calves.

While the dietary supplementation of n-3 fatty acids, especially during the critical stage that is dairy cows’ peripartum period, is, in general, expected to enhance reproductive performance, the impact of its supplementation appears to be dependent on the body condition scores of cows during the drying period, the severity of the negative energy balance, and the amount of fat in the basic feed ration. The observed positive impact of n-3 fatty acids’ supplementation increases the concentration of n-3 FA, in particular EPA and DHA in plasma phospholipids, and positively affects estrous cyclicity, as well as increases the follicle number during ovum pickup, increases the percentage of oocytes that develop into blastocysts, improves oocyte cleavage, shortens the time of the first estrus, and improves the conception rate. However, due to the diversity of the sources of n-3 used in the studies (flaxseed oil, fish oil, algae), it is difficult to estimate the dose of n-3 FA that most effectively improves the reproduction indices of cows. Based on the presented results, the effective and beneficial dose of n-3 FA seems to be between 30 and 70 g/head/day prepartum and between 50 and 150 g/head/day postpartum. However, this aspect requires further study as the recommendations regarding the dose are not clear.

The results of presented studies also prove that, from the prenatal stages, fatty acids and their metabolites play a key role in the processes of cell growth, differentiation, and regulatory response between metabolic and neuroendocrine environments. The unbalanced, insufficient, or excessive fatty acid supplementation of cows’ diets in the early stages of pregnancy and during fetus development may affect both the metabolic and nutritional programming of the offspring. The presence of the polyunsaturated fatty acids of the n-3 family in the calves’ ration affects not only the performance of calves but also the immune response, antioxidant status, and overall metabolism of the future adult cow. Further investigations are required, though, to explain the mechanisms underlying the effect of n-3 fatty acids on maternal–offspring relations in building future immunity.

## Figures and Tables

**Table 2:** Effectiveness of n-3 fatty acid supplementation in calf nutrition—summary.

Experimental Animals	Experiment Design	Share of Fatty Acids in the Ration/Fat Supplement	Effects of Supplementation	Source
48 Holstein calves aged 2–5 days	Calves were randomly assigned to 1 of 3 diets (16 calves/diet): milk replacer with 17% pork fat, milk replacer with 15% pork fat and 2% DM flaxseed oil (flax), or milk replacer with 15% DM pork fat and 2% DM fish oil (fish) Experiment duration: 56 days	Control: n-3—1.7% n-6—22.4% n-6/n-3—12.8% Flax: n-3—12.5% n-6—21.3% n-6/n-3—1.7% Fish: n-3—6.1% n-6—21.4% n-6/n-3—3.5%	FLAX group: IL-1β ↓ IL-8 ↓ Osteopontin ↓ FISH group: TNF-α ↓	[196]
54 calves of the Holstein Friesian breed at 6 days old	Animals were divided into 3 groups (n =18 calves each): control, the group receiving milk replacer + beta-carotene (25 mg/calf/day), and the group receiving milk replacer + addition of liver oil suspension, as a source of n-3 (5 g/calf/day) Experiment duration: 54 days	Liver oil: SFA—15.2% MUFA—47.5% PUFA—31.4% n-3—27.2%	IgG ↑ ALT ↑ AST ↓ ALP ↑ LDH-L ↑ UREA ↓ CREA ↓ Glucose ↓ Cholesterol ↑ Occurrence of diarrhea ↓ Diseases of the upper tract ↓	[193]
15 Holstein Friesian calves at 7 days of age	Animals were divided into 3 groups (n=18 calves each): control, the group receiving milk replacer + beta-carotene (25 mg/calf/day), and the group receiving milk replacer + addition of liver oil suspension, as a source of n-3 (5 g/calf/day) Experiment duration: 54 days	Canola oil: * SFA—45.79% MUFA—24.06% PUFA—28.77% n-3—13.36% Fish oil: * SFA—33.37% MUFA—40.73% PUFA—24.96% n-3—11.23%	Fish oil (source of n-3) group: Hepatoglobins ↓ IL-1β ↓ TNF-α ↓	[200]
30 Holstein calves aged 1–4 days	Animals were randomly assigned to be fed a milk replacer with an n-6:n-3 FA ratio of 40:1 or 6.5:1 The PUFA ratio in the milk replacer was adjusted by including 1% flaxseed oil and 1% algae oil (as a share in fat content in the diet) Experiment duration: 25 days	Control: SFA—63.7% UFA—36.3% n-3—0.16% n-6—6.49% n-6:n-3—40.6% Experimental diet: SFA— 62.6% UFA—37.4% n-3—1.04% n-6 —6.76 n-6:n-3—6.5%	Acute phase proteins ↑ Haptoglobin ↑ Amyloid A ↑	[194]
40 Holstein Friesian calves at 8.6 days of age	Animals were assigned to 4 experimental groups: control, milk replacer + addition of 9 g of DHA-rich algae; milk replacer + addition of 9, 18, 27 g of DHA-rich algae Experiment duration: 49 days	Algae: * SFA—63.57% MUFA—1.24% PUFA—34.47% n-3—33.69%	IL-1β ↓ TNFα ↓ IgG ↓	[201]

* calculated based on the reported fatty acid profile: ↑ increase; ↓ decrease. Explanations: IL-1β—pro–inflammatory interleukin; IL-8—interleukin 8; TNF-α—tumor necrosis factor-α; DM—dry matter; SFA—saturated fatty acids; MUFA—monounsaturated fatty acids; PUFA—polyunsaturated fatty acids; FA—fatty acid; DHA—docosahexaenoic acid; n-3—omega-3 fatty acid; n-6—omega-6 fatty acids; IgG—immunoglobulin G; ALT—alanine aminotransferase; AST—alanine aminotransferase; ALP—alkaline phosphatase; LDH-L—lactate dehydrogenase; CREA—creatinine.

## Data Availability

The data presented in this study are available in this paper.

## Abbreviations

ALT	alanine aminotransferase
AST	aspartate aminotransferase
ATP	adenosine triphosphate
BHB	beta-hydroxybutyrate
C12:0	lauric acid
C14:0; MA	myristic acid
C16:0; PA	palmitic acid
C18:0; STA	stearic acid
C18:1	oleic acid
C18:1; TVA	vaccenic acid
C18:2; LA	linoleic acid
C18:3 n-3; ALA	alpha-lipoic acid
C18:3; LNA	linolenic acid
C20:4; AA	arachidonic acid
C20:5 n-3; EPA	eicosapentaenoic acid
C22:6 n-3; DHA	docosahexaenoic acid
CAT	catalase
CD62	selectin P
CL	corpus luteum
CNS	central nervous system
COX-2	enzyme cyclooxygenase-2
CPT-I	carnitine palmitoyltransferase-I
DNMT	DNA methyltransferase
EFA	essential fatty acids
FA	fatty acids
FABP	intracellular FA-binding proteins
FATP	fatty acid transporter proteins
FFAR	free fatty acid receptor
FFAs	free fatty acids
FLX	flaxseed
FO	fish oils
GPx (GSH-Px)	glutathione peroxidase
IFN	interferon
IFN-τ	interferon-tau
Ig	immunoglobulins
IgA	immunoglobulin A
IgE	immunoglobulin E
IGF-1	insulin-like growth factor 1
IgG	immunoglobulin G
IgM	immunoglobulin M
IL	interleukin
IL-1β	pro-inflammatory interleukin
IL-4	interleukin-4
IL-8	interleukin-8
ISGs	interferon-induced genes
IVM	in vitro maturation medium
LC-PUFA	long-chain polyunsaturated fatty acids
LF	lactoferrin
LH	luteinizing hormone
LPS	lipopolysaccharides
Lz	lysozyme
MDA	lipid peroxide
MII	oocyte into metaphase II
MONO	monocytes
MR	milk replacer
mRNA	messenger RNA
MUFA	monounsaturated fatty acid
N-3 PUFA	omega-3 fatty acids
NEB	negative energy balance
NEFA	non-esterified fatty acids
NFκB	nuclear factor kappa B
NK	natural killer cells
NL	neutral lipids
OCC	oocyte–cell complex
OSi	oxidative status index
PBL	blood leukocytes
PBMC	peripheral blood mononuclear cells
PG	prostaglandin
PGFM	prostaglandin F2 alpha metabolite
PGHS	prostaglandin endonadoxide synthase
PL	phospholipids
PPARs	peroxisome proliferator-activated receptors
PTGS2	prostaglandin-endoperoxidase 2
PUFA	polyunsaturated fatty acid
RELA	v-rel avian reticuloendotheliosis viral oncogene homolog A
RONS	reactive oxygen and nitrogen species
SFA	saturated fatty acid
SOD	superoxide dismutase
TAG	triacylglycerol
TAS	total antioxidant capacity
TG	triglycerides
Th1	T helper 1
TNF-α	tumor necrosis factor-α
TXA2	thromboxane A2
UFA	unsaturated fatty acids
VLDL	very low-density lipoproteins
WBC	leukocytes
α-LG	α-lactalbumin
β-LG	β-lactoglobulin
